# Advancing learning-oriented assessment (LOA): mapping the role of self-assessment, academic resilience, academic motivation in students’ test-taking skills, and test anxiety management in Telegram-assisted-language learning

**DOI:** 10.1186/s40468-023-00230-8

**Published:** 2023-04-06

**Authors:** Fidel Çakmak, Sayed M. Ismail, Samaneh Karami

**Affiliations:** 1 Department of Foreign Language Education, Alanya Alaaddin Keykubat University, Antalya, Turkey; 2 College of Humanities and Sciences, Prince Sattam Bin Abdulaziz University, Al-Kharj, Saudi Arabia; 3 Department of English Language Teaching, Cardiovascular Diseases Research Center, School of Medicine, Guilan University of Medical Sciences, Rasht, Iran

**Keywords:** Self-assessment, Academic resilience, Academic motivation, Test-taking skills, Test anxiety management, Structural equation modeling

## Abstract

Some impediments in language learning may have a detrimental impact on learners’ actual performance on the test and lead to anxiety and demotivation. Language achievement is influenced by self-assessment (SA), academic resilience (AR), academic motivation (AM), and test-taking skills (T-TS) among other factors. Considering the relevance of these factors in language achievement, the current investigation aims to delve into the probable interactions of SA, AR, AM, T-TS, and test anxiety (TA) management among English as a foreign language (EFL) learners. A model was devised and evaluated using confirmatory factor analysis (CFA) and structural equation modeling (SEM) to achieve this objective. This research collected 512 by distributing online questionnaires to fifteen approved private institutions which applied Telegram-based language learning. The study findings reflected that SA, AR, and AM could predict EFL learners’ T-TS. It was also confirmed that SA, AR, and AM modulated EFL learners’ TA. The implications of the study are presented and accompanied by some future research proposals as well as instructional consequences.

## Introduction

Several factors impact the decisions that educators and test developers must make to establish and administer an effective education and assessment program. Both emotive and cognitive characteristics of the learners are critical in successful language testing. Practically speaking, learning-oriented evaluation in the classroom operates reasonably well to evaluate language learners’ language progress and observe their mental and emotional well-being (Bourke & Mentis, 2007). During the COVID-19 pandemic crisis, which led to school and university closings and prompted the adoption of distance learning modalities, social media usage is increasingly prominent. Online programs and social media allow for two-way communication between professors and their students. Mobile-assisted language learning (MALL) is employed as a means to provide complementary educational opportunities during the COVID-19 pandemic shutdown. MALL is employed as a means of providing additional educational opportunities beyond those made available during the COVID-19 pandemic shutdown (Azizi et al., 2022; Barrot, 2021). Telegram is a social network tool that has become very popular because of its ease of access, its simplicity of exchanging ideas and input, its capacity for holding various files, and its potential for managing online courses. Telegram is compatible with the operating systems used by Android, iOS, and Windows Phone, as well as Mac and Windows computers. Also, the Telegram app may be accessed concurrently from numerous devices and may open new perspectives in language-oriented assessment (LOA). The goal of LOA is to provide a systematic approach to language acquisition by using formal and informal evaluation to aid in the planning of learning, monitor progress, pinpoint areas for development, and offer quantifiable results. The framework that LOA offers makes it easy to combine in-class assessments, self-assessment, and less qualitative forms of student observation. It aids in course planning and making ensuring that in-class and extracurricular activities all work together to assist each student to reach his or her own goals (Bachman, 2015).

There is a possibility that instructors of foreign languages and language learners alike might experience a negative impact on their mental health as a result of the integration of technology into language teaching and learning. This new challenge inspires educators and researchers to apply effective strategies to help teachers and learners achieve educational perspectives. This new adventure motivates researchers and educators to employ solutions that assist instructors and students in achieving their educational goals. SA intended to activate learners to evaluate their learning progress and accomplishments (Wrigglesworth, 2019). More precisely, learners are directly involved in the processes of their assessments, hence providing a clearer picture of how pupils perform and where they may be experiencing difficulty (Bachman, 2015). AR refers to students’ abilities to successfully control hardships, difficulties, stresses, and setbacks in the academic setting (Martin, 2006). AR supports language learners to overcome language learning obstacles and develop L2. The development of AR requires a positive attitude, the will to improve, and the ability to learn from mistakes (Ayoobiyan & Rashidi, 2021).

Another student-attributed construct is motivation, which is viewed as a stimulant that influences the conduct of humans (Dörnyei, & Ottó, 1998). In the educational arena, AM refers to their dedication to the educational processes (Hiver & Al-Hoorie, 2020). Regarding language learning, Peng (2021) defined AM as the amount to which they seek to acquire a new language and their involvement in the learning process. It is important to consider this point that students’ psychological balance may be altered by the spread of new technologies, their assimilation into the field of education, and the introduction of several new difficulties. Learners are more likely to suffer anxiety when they have the perception that they are unable to modify the outcomes of a situation (Namaziandost et al., 2022a, 2022b; Oteir and Al-Otaibi, 2019). Changes in the form of the test may lead to test takers’ anxiety. Anxiety over taking exams can have a detrimental impact on academic performance by reducing one’s ability to concentrate, organize their ideas and thoughts, effectively manage their time during examinations, and comprehend the topics and questions being asked.

Even though SA, AM, and AR have been independently verified to assist learners in better managing their T-TS and TA, which consequently results in enhanced educational outcomes for the students, there has never been a study done to investigate the links between them. This research gap is also glaringly obvious in the existing body of scholarly work on the subject of the contributions that the Telegram app, which can be thought of as a type of social media platform, makes to language acquisition. In light of the aforementioned research deficiencies and the significance of the learners’ attributed constructs (specifically, SA, AM, and AR) in terms of their T-TS and TA, this research sought to explore the contribution of SA, AM, AR to T-TS, and TA management. Holding these standpoints into consideration led to the formulation of the following research questions:

1. Can EFL learners’ SA provide insight into their T-TS in Telegram-assisted-language learning?
2. Can EFL learners’ SA provide insight into their TA in Telegram-assisted-language learning?
3. Can EFL learners’ AR provide insight into their T-TS in Telegram-assisted-language learning?
4. Can EFL learners’ AR provide insight into their TA in Telegram-assisted-language learning?
5. Can EFL learners’ AM provide insight into their T-TS in Telegram-assisted-language learning?
6. Can EFL learners’ AM provide insight into their TA in Telegram-assisted-language learning?

In this respect, the following null hypothesis are proposed:

- H01: EFL learners’ SA does not provide insight into their T-TS in Telegram-assisted-language learning.
- H02: EFL learners’ SA does not provide insight into their TA in Telegram-assisted-language learning
- H03: EFL learners’ AR does not provide insight into their T-TS in Telegram-assisted-language learning.
- H04: EFL learners’ AR does not provide insight into their TA in Telegram-assisted-language learning
- H05: EFL learners’ AM does not provide insight into their T-TS in Telegram-assisted-language learning
- H06: EFL learners’ AM does not provide insight into their TA in Telegram-assisted-language learning

**Fig. 1:**
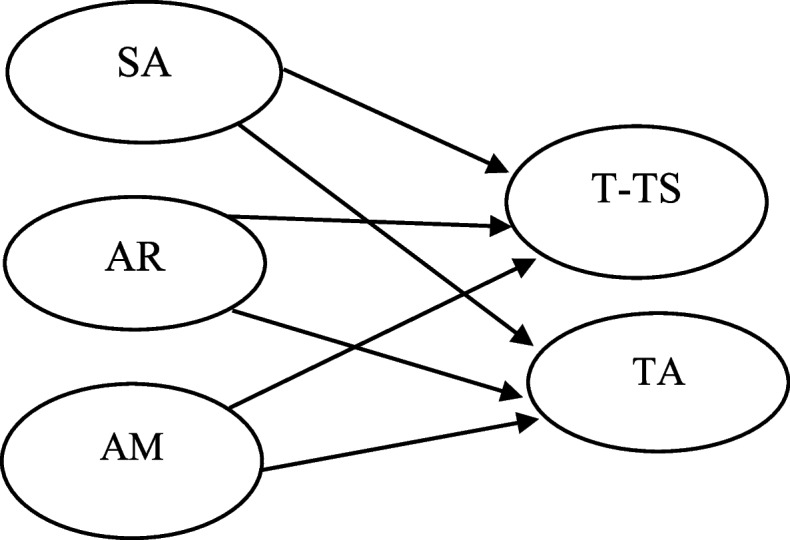
Theoretical structural equation model

As a response to these inherent gaps, a model was developed to illustrate the probable linkages between SA, AR, AM, T-TS, and TA. This model was developed based on pertinent theories and contemporary literature (see Fig. 1). Using CEF and SEM, the suggested model was then assessed.

### Overview

The term “assessment” encompasses an array of methods that are used to analyze and draw conclusions about the advancement that pupils have made in their studies (Bachman, 2015). Many alternative ways have been presented to accurately and thoroughly evaluate the progress that learners have made and to prepare for future activities. Learners’ active engagement in their own evaluation is referred to as SA, which is distinct from both teacher and peer assessment. SA was described by Bachman et al. (2010) as the “examination or evaluation of oneself or one's behaviors, attitudes, or performance.” In this regard, Andrade (2019) argues that SA is a kind of assessment that emphasizes intellectual abilities, conceptual understanding, and conscience learning. Learners’ cognitive abilities are not the only thing that gets impacted when they practice SA; their emotional well-being does, too, since their techniques have an effect on it (Punpromthada et al., 2022). Pupils improve their capacity to think critically and make well-considered decisions by participating in self-evaluation activities. These activities additionally assist pupils to become more adept at overcoming educational challenges (Al-Mamoory & Abathar Witwit, 2021). Also, it was demonstrated that several aspects may be responsible for setting the ethos of SA among the trainees. Notably, metacognitive strategies (Wei, 2020), and CT (Zhang, 2021), as well as self-efficacy skills (Zheng et al., 2022), emotion management (Heydarnejad et al., 2022), are among the characteristics that enhance students’ engagement in their SA. Along with this vein of research, Jahara et al. (2022) found evidence that the coping style of students had a favorable influence on both their SA and their ability to handle stress. The recent study by Ismail and Heydarnejad (2023) confirmed that SA and evaluation apprehension could predict EFL learners’ personal best goals and self-efficacy beliefs.

Resilience has been conceptualized as the ability to sustain normal development and make positive adaptations while facing significant adversity (Fletcher & Sarkar, 2003). Martin and Marsh (2009) refer to AR as an inherently asset-oriented and aspirational method that helps students to overcome problems and adversities in educational environments. As Campbell Sills et al. (2006) noted, AR is a multidimensional construct, and various factors play a crucial part in its construction and growth. In addition to particular talents like active problem-solving and personality characteristics, these factors encompass temperament and personality as well (Campbell Sills et al., 2006). Resilient students attempt to overcome obstacles and are more likely to be successful in completing challenging projects (Tamannaeifar & Shahmirzaei, 2019). AR gives students the courage to take chances, which reduces their fear of dropping out of school and failing their subjects (Rojas, 2015). Furthermore, AR helps students to handle anxiety and despair caused by language lessons (Khadem et al., 2017). The increasing focus on AR in recent years was mirrored in the review of relevant literature. Rahat and Ilhan (2016), for instance, investigated how pursuits like storytelling, discovery learning, and problem-solving may contribute to the development of AR. In their research, Rudd et al. (2021) noted that AR is a dynamic and supporting construct that molds supportive adaptation to overcome obstacles to beneficial development. In addition, Karabıyık (2020) and Namaziandost et al. (2023) offered evidence that reflection and help-seeking are both significant in the progress of AR.

AM is an essential component in the psychological health of a student and has a significant impact on how they behave. This idea pertains to the learners’ interest in the academic topics they are studying, which shapes their personality, perspectives toward education, and their efforts despite the challenges they confront (Al-Hoorie et al., 2022). State motivation and trait motivation are the two categories that Dörnyei et al. (2016) use to describe student AM. As Alamer and Almulhim (2021) as well as Heydarnejad et al. (2018) put it, learners’ tendencies toward a certain topic may be captured by their state motivation. According to Dörnyei (2020), the term trait motivation refers to the learners’ overall attitude toward the learning process. According to the research of Ushioda (2016), trait motivation is consistent over time, but state motivation is fluid and may change over time. Static motivation may be influenced by a number of different elements, including the learning environment and the content of the course, as well as the personalities of the instructors and their relationships with the students (Martin, 2013).

The self-determination theory (SDT), which was first proposed by Deci and Ryan (1985), is now the dominant hypothesis in the field of AM explanation. The SDT postulated that there are three distinct aspects of motivation, namely amotivation, inner motivation, and extrinsic motivation. When students have a strong level of motivation, they do not pause in their learning but instead excitedly follow the processes (Deci & Ryan, 2020). AM protects learners even in the face of adversity they may experience along the path to learning (Howard et al., 2021). In this respect, Peng (2021) discovered that EFL instructors’ communication styles might encourage learners’ active involvement in their learning. Moreover, Cao (2022) evinced the roles that AM and L2 pleasure play in moderating the relationship between learners’ desire to communicate in their second language.

TA may be experienced by learners before, during, and after the test. Horwitz et al. (1986) introduced a model of anxiety in foreign language learning that included three components (i.e., communication apprehension, test anxiety, and fear of negative evaluation). The first factor, known as communication apprehension, relates to the nervousness felt while dealing with other people, engaging in verbal communication, or having difficulties understanding what is being heard. The second component, test anxiety, is the dread that comes with the prospect of doing poorly on an assessment. Uneasiness about the assessments of other people and avoiding situations that might lead to the appraisal of others in an unfavorable light are the focus of the third dimension, which is titled fear of negative evaluation. There is a possibility that students’ anxiety might be triggered by both internal and external variables (Alamer & Almulhim, 2021). The attentional control theory (ACT) gives a rationale for the reason why anxiousness is detrimental to the academic performance of students (Eysenck et al., 2007). ACT argues that anxiety inhibits attentional control by accumulating threat-related inputs. According to Cassady (2010), the word academic anxiety is a broad term that applies to a range of worries that students feel in the context of their academic endeavors. According to him, the anxiety that pupils experience hinders their educational outcomes. The research conducted by Khajavy et al. (2021) concluded that language learners’ anxiety hinders their willingness to communicate in language classes. Similarly, Fathi et al. (2021) demonstrated that anxiety and grit are a detriment in predicting EFL learners’ achievement in oral communication.

The COVID-19 pandemic has interrupted education in many countries; thus, as an emergent reaction, several governments have prioritized the implementation of virtual instructional strategies. During the COVID-19 outbreak, online courses and social media facilitated two-way communication between pupils and their instructors (Peng, 2021). In recent years, in addition to online development, MALL for learning English with networking technologies has attracted considerable interest. MALL has been developed to facilitate the language acquisition of students via activities conducted on mobile gadgets at any moment and place (Wrigglesworth, 2019). Social media served as a supplement to the teaching–learning process and as a platform for the dissemination of instructional materials, engagement, and assessment during the COVID-19 pandemic. The application known as Telegram quickly rose to be one of the most popular choices among those that may be utilized on mobile devices such as smartphones.

Telegram is compatible with the operating systems used by Android, iOS, and Windows Phone, as well as Mac and Windows devices. Furthermore, the Telegram app may be used concurrently from a number of different devices (Chen & Jia, 2020). When the benefits of the Telegram app are considered, every educational field, in particular the study of languages, has observed the program’s application and the boosting impacts it has had. In this regard, Abu-Ayfah (2019) found that the Telegram app aided EFL students in the process of vocabulary acquisition. Similarly, Shirinbakhsh and Saeidi (2018) provided evidence that confirmed the beneficial impact of Telegram on the improvement of students’ reading skills during IELTS preparation sessions. When compared with their peers in EG, the students in CG demonstrated superior performance.

Connectivism theory provides support for the implementation of learning strategies that make use of social media and other online connections (Greenhow & Lewin, 2016). This theory has its foundations in distributed learning (Siemens, 2004), and it takes into account the role that digital means of interaction play in the educational process. The fundamental tenet of the connectivism philosophy is that ideas, theories, and information ought to be linked to one another effectively. Connectivism also has its roots in self-organization, and its proponents hold the belief that education delivered through social media platforms increases students’ capacity for self-reliance and autonomy (Greenhow & Lewin, 2016). The constructivism theory also provides evidence for the contribution that social media might make to the learning process (Kelm, 2011). Increases in social connections, online learning, cooperation, and academic motivation are brought about with the assistance of platforms provided by social media, which helps to ensure that a constructivist learning environment is maintained (Manca, 2020).

## Methodology

### Participants and design

A total of 512 students enrolled in fifteen private English language institutions took part in this survey: 263 men and 249 women. Ages between 21 and 37 took part in this study who learned English at advanced level via Telegram. The selection of the participants was carried out using procedures that included either sampling based on convenience or random sampling.

## Materials

### The Core of Self-assessments Questionnaire (CSAQ)

The CSAQ, which was created by Judge et al. (2003), was used to evaluate the students’ SA at the institution. On this scale, there are 12 different items, each with a Likert scale rating from 1 to 5: strongly disagree (1) to strongly agree (5). The scores that the pupils achieved on this scale ranged anywhere from 12 to 60. A favorable self-assessment was expressed by high scores on this scale. The current investigation found that the CSEQ had adequate reliability, with a value of 0.897 for its coefficient of reliability.

### The Academic Resilience Scale (ARS)

To evaluate the AR, the ASR was utilized that Kim and Kim (2016) developed. In this scale, there are a total of 26 items, each with a Likert value ranging from one to five. These items are categorized as follows: subjective happiness (9 items), empathy (7 items), sociability (3 items), perseverance (4 items), and self-regulation (2 items). The results displayed that the SRS has an acceptable reliability that ranges from 0.837 to 0.892.

### The Test-Taking Skills Scale (T-TSS)

Test-taking skills of participants were evaluated utilizing Dodeen’s T-TSS, which was created to measure those abilities (2008). Before-Test, Time Management, During-Test, and After-Test are the subscales that are comprised of a total of 31 items that make up this scale. If a student has a high score on the TSS, it shows that they have acceptable test-taking skills. The internal consistency of this scale was acceptable, ranging from 0.858 to 0.901.

### The Westside Test Anxiety Scale (WTAS)

Driscoll (2007) designed the WTAS, which consists of ten items, each of which uses a Likert answer scale. The participants were instructed to verify their replies on a scale ranging from “very, always true” to “not at all, never true.” It provides a score for overall anxiety as well as measures the impairments caused by anxiety. It does this by using six items to assess Incapacity and four items to assess Worry and Dread, both of which intrude on concentration. In this investigation, Cronbach’s alpha findings of 0.865 show considerable consistency.

### The Achievement Motivation Scale (AMS)

The AMS (Vallerand et al., 1992) was used to assess AM among the participants. AMS was established based on the self-determination theory, and it has 7 dimensions: internal motivation toward knowledge, and accomplishments, to experience stimulation, external motivation as introjected, identified regulations, and amotivation. A total of 28 elements are included on this Likert scale that ranges from 1 to 7 (strongly agree to strongly disagree). The full scores on this scale range from 28 to 196. Cronbach’s alpha was calculated for this research, and the findings showed that it had substantial dependability.

### Data collection procedures and analysis

This research was carried out using a web-based platform, and it began in October of 2022 and continued through December of the same year. The participants were requested to fill out a Google Forms questionnaire that included the CSAQ, ARS, TSS, WTAS, and AMS. To ensure that no data were overlooked, the structure of the electronic survey required that every section of the electronic survey form be inextricably linked to one another. Thus, each part should be necessarily linked. There were 512 completed forms received, with a return rate of 87.9%. The Kolmogorov–Smirnov test was utilized to examine the data’s normal distribution. Due to the normal distribution of the data, CFA and SEM using LISREL 8.80 were utilized.

## Results

This part presents the results of the statistical analysis. The following table provides descriptive information regarding the selected variables, namely SA, AR, AM, T-TS, and TA.

As Table 1 presented, the mean score of SA was (*M* = 40.453, *SD* = 10.178). Among the subcomponents of AR, Perceived Happiness (*M* = 59.965, *SD* = 8.290) received the highest mean score. Regarding AM, the mean score was (*M* = 116.119, *SD* = 10.395). Furthermore, During-Test was the highest (*M* = 28.598, *SD* = 7.391) among T-TS. In the final variable, TA, Incapacity was different (*M* = 20.402, *SD* = 5.496). Then, the normality of the data was assessed and reported in Table 2.

**Table 1:** Descriptive statistics

	*N*	Minimum	Maximum	Mean	Std. deviation
The Core of Self-assessments Questionnaire (SAQ)	512	12	60	40.453	10.178
Perceived happiness	512	9	45	29.852	7.720
Empathy	512	10	35	24.064	5.952
Sociability	512	3	15	10.555	3.026
Persistence	512	4	20	13.549	3.777
Self-regulation	512	2	10	6.822	2.227
The Academic Resilience Scale (ARS)	512	30	125	84.842	18.965
Achievement Motivation Scale (AMS)	512	72	196	116.119	10.395
Before-Test	512	11	40	28.826	7.724
Time Management	512	8	40	29.318	7.501
During-Test	512	13	40	28.598	7.391
After-Test	512	11	35	26.373	4.124
Test-Taking Skills Scale (T-TSS)	512	54	155	113.115	23.615
Incapacity	512	6	30	20.402	5.496
Worry and Dread	512	6	20	15.057	3.141
Test Anxiety Scale (TAS)	512	12	50	35.459	8.255

**Table 2:** The results of Kolmogorov–Smirnov test

Instrument	Subfactors	Kolmogorov–Smirnov Z	Asymp. sig. (2-tailed)
The Core of Self-assessments Questionnaire (SAQ)	Perceived happiness	0.430	0.993
	Empathy	0.608	0.854
	Sociability	0.824	0.505
	Persistence	0.561	0.912
	Self-regulation	1.172	0.128
The Academic Resilience Scale	(ARS)	0.775	0.585
Achievement Motivation Scale (AMS)	Before-Test	0.926	0.358
	Time Management	1.152	0.140
	During-Test	0.918	0.368
	After-Test	1.136	0.152
Test-Taking Skills Scale (T-TSS)	Incapacity	0.777	0.581
	Worry and Dread	1.305	0.066
Test Anxiety Scale	TAS	0.677	0.750

Based on what Table 2 presented, the data were normally distributed, and parametric methods are logical to be used. Therefore, CFA and SEM were considered to gauge the structural relationships between SA, AR, AM, T-TS, and TA by the LISREL 8.80 statistical package. To assess the model fit, the chi-square magnitude, the root-mean-squared error of approximation (RMSEA), the comparative fit index (CFI), and the normed fit index (NFI) were utilized (Jöreskog, 1990).

**Fig. 2:**
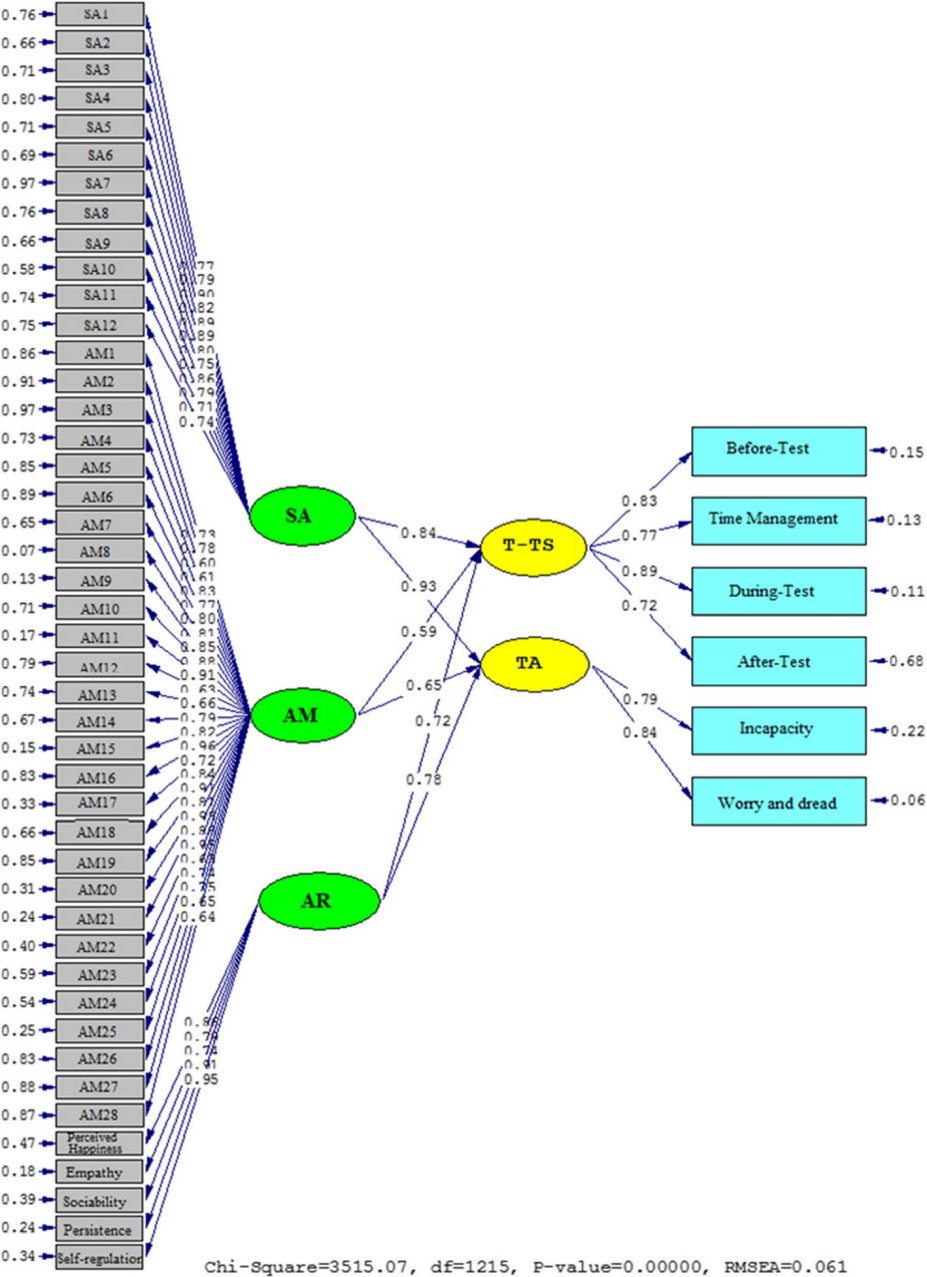
A graphical representation of the values of the path coefficients for the relationships between SA, AR, AM, T-TS, and TA (model 1)

**Fig. 3:**
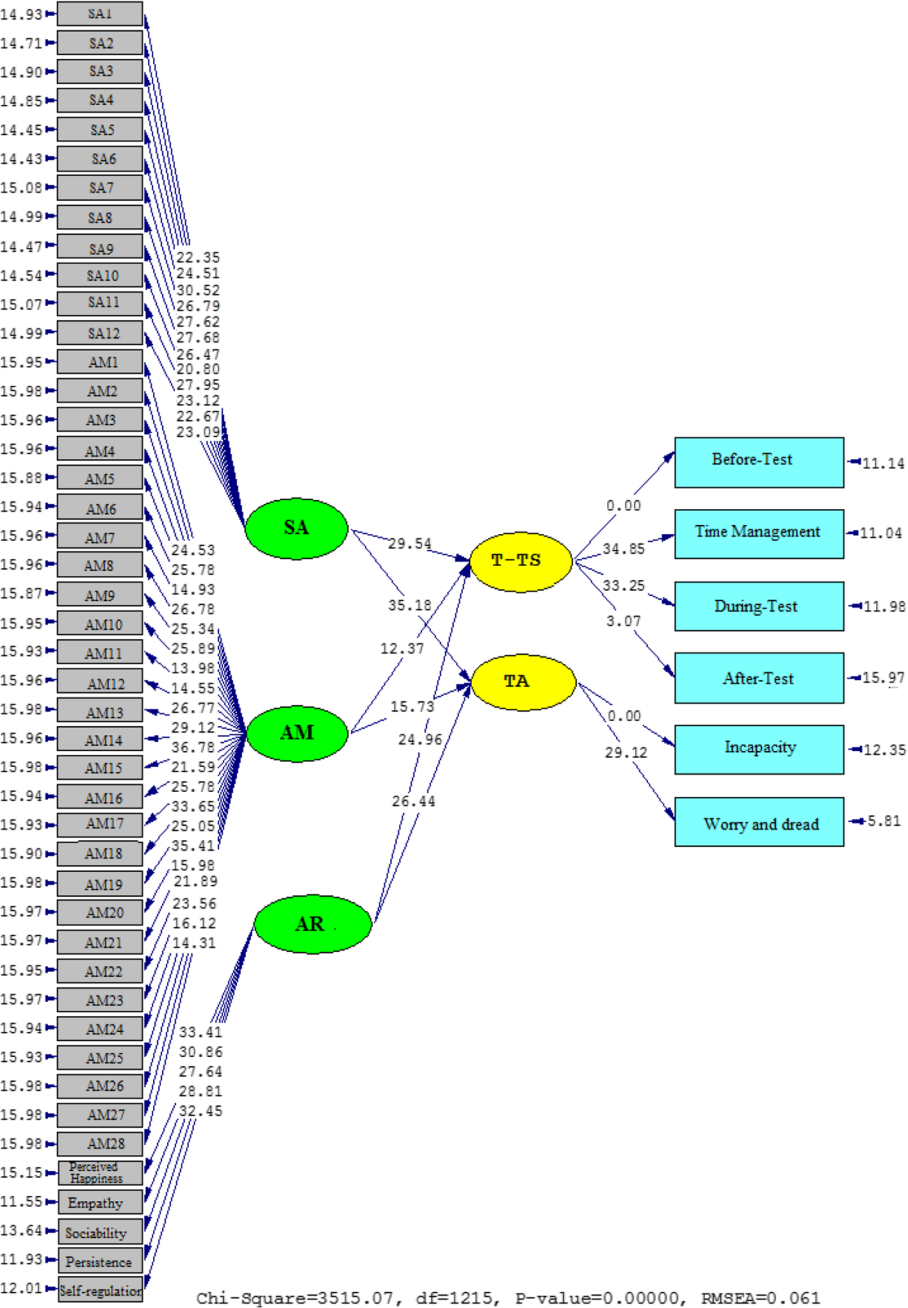
T significance levels for path coefficients (model 1)

The causal relationships among the variables are portrayed in Figs. 2 and 3. They show that SR, CT, AER, SE, and SD are negatively related. The influence of SA on T-TS (*β* = 0.84, *t* = 29.54) and TA (*β* = 0.93, *t* = 35.18) was positive. The effect of AR on T-TS (*β* = 0.72, *t* = 24.96) and TA (*β* = 0.78, *t* = 26.44) was significant. The same was true about the impact of AM on T-TS (*β* = 0.59, *t* = 12.37) and TA (*β* = 0.65, *t* = 15.73).

The data in Table 3 indicate that the chi-square/df ratio (2.926), the RMSEA (0.070), GFI (0.955), NFI (0.973), and CFI (0.948) met the acceptable fit standards for model 1. Moreover, Table 3 summarizes that the model fit indices related to model 2 were all acceptable: the chi-square/df ratio (2.947), the RMSEA (0.062), GFI (0.925), NFI (0.938), and CFI (0.956).

**Table 3:** Model fit indices

Fitting indexes	${\varvec{\chi}}2$	$\mathbf{d}\mathbf{f}$	${\varvec{\chi}}2/{\mathbf{d}}{\mathbf{f}}$	RMSEA	GFI	NFI	CFI
Cut value			< 3	< 0.1	> 0.9	> 0.9	> 0.9
Model 1	3515.07	1215	2.893	0.061	0.955	0.973	0.948
Model 2	10,457.61	3548	2.947	0.062	0.925	0.938	0.956

**Fig. 4:**
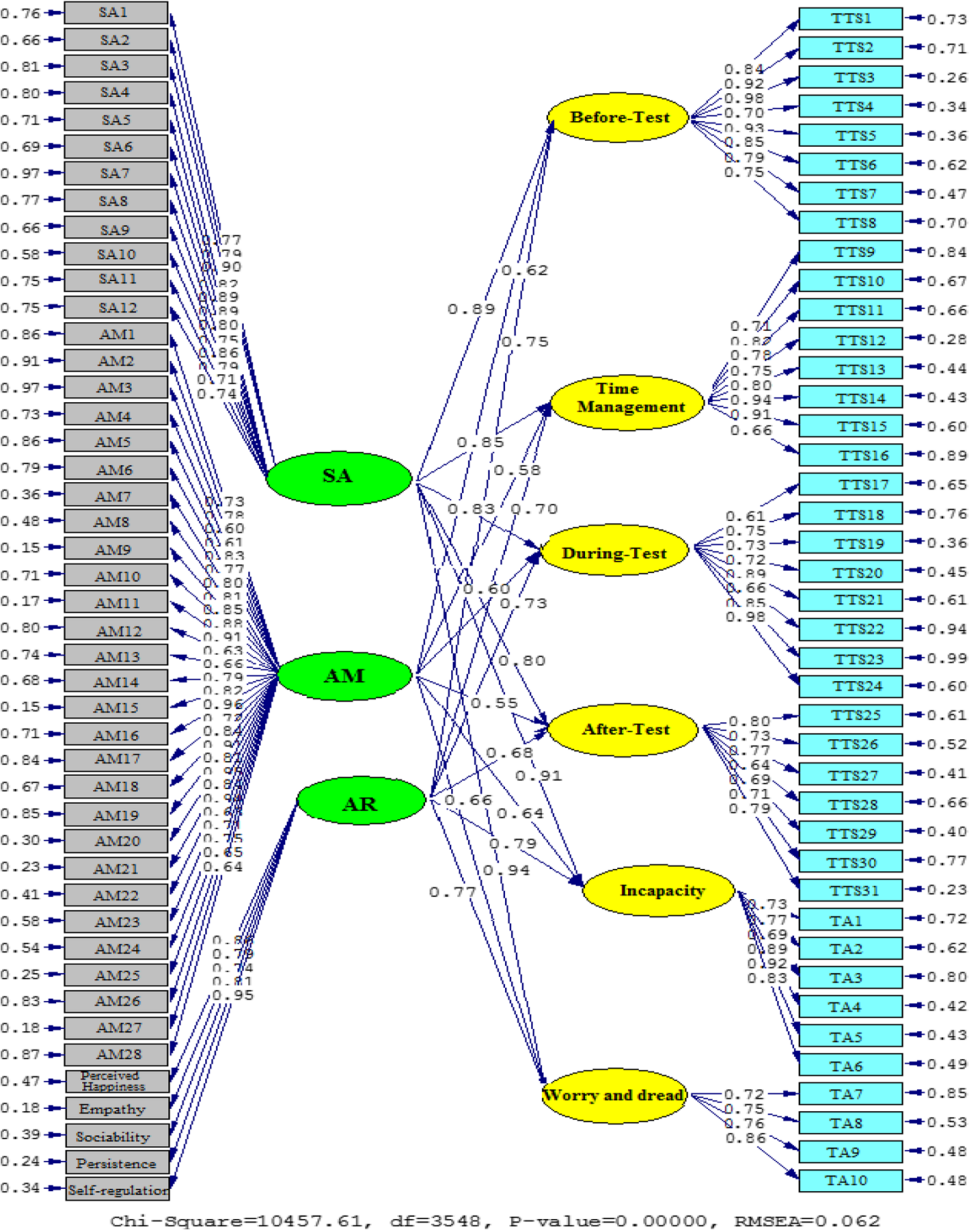
A graphical representation of the values of the path coefficients for the relationships between SA, AR, AM, T-TS, and TA subscales (model 2)

**Fig. 5:**
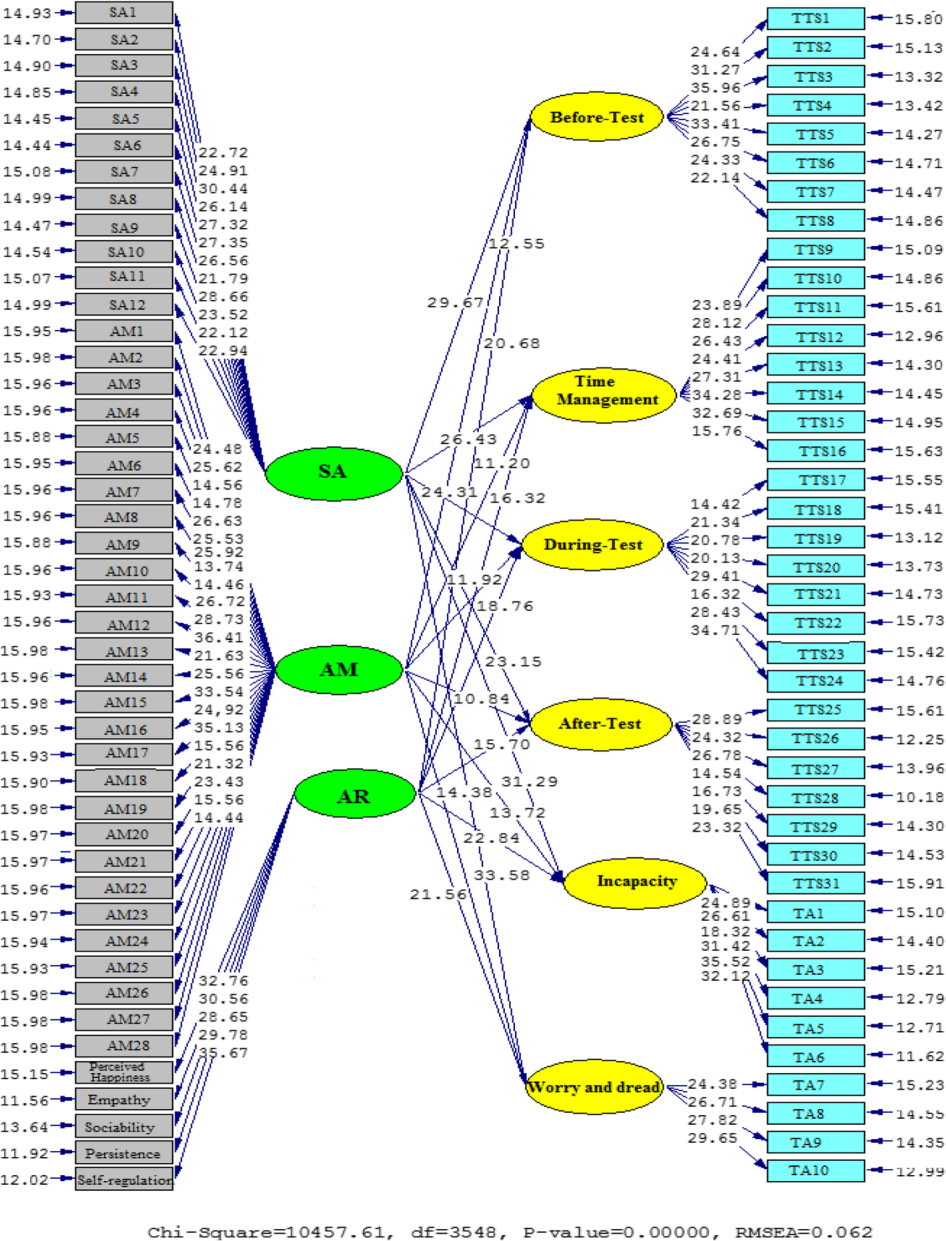
T significance levels for path coefficients (model 2)

In model 2 which is illustrated via Figs. 4 and 5, a graphical representation of the values of the path coefficients for the relationships between SA, AR, AM, T-TS, and TA subfactors is displayed. Considering SA and T-TS and TA subfactors, the results are as follows: SA on Before-Test (*β* = 0.89, *t* = 29.67), Time Management (*β* = 0.85, *t* = 26.43), During-Test (*β* = 0.83, *t* = 24.31), After-Test (*β* = 0.80, *t* = 23.15), Incapacity (*β* = 0.91, *t* = 31.29), and Worry and Dread (*β* = 0.94, *t* = 33.58). The association between AR, TS, and TA subfactors is as follows: AR and Before-Test (*β* = 0.75, *t* = 20.68), Time Management (*β* = 0.70, *t* = 16.32), During-Test (*β* = 0.73, *t* = 18.76), After-Test (*β* = 0.68, *t* = 15.70), Incapacity (*β* = 0.79, *t* = 22.84), and Worry and Dread (*β* = 0.77, *t* = 21.56). Additionally, AM is linked with TS, and TA subfactors are as follows: Before-Test (*β* = 0.62, *t* = 12.55), Time Management (*β* = 0.58, *t* = 11.20), During-Test (*β* = 0.60, *t* = 11.92), After-Test (*β* = 0.55, *t* = 10.84), Incapacity (*β* = 0.64, *t* = 13.72), and Worry and Dread (*β* = 0.66, *t* = 14.38).

As the final step, a Pearson product-moment correlation was run to address the correlation between SA, AR, AM, T-TS, and TA subfactors.

Table 4 indicates that the SA, AR, and AM are strongly and positively linked with T-TS and TA subfactors: SA and Before-Test (0.924), SA and Time Management (0.875), SA and During-Test (0.851), SA and After-Test (0.824), SA and Incapacity (0.932), SA as well as Worry and Dread (0.955); AR and Before-Test (0.776), AR and Time Management (0.724), AR and During-Test (0.756), AR and After-Test (0.706), AR and Incapacity (0.815), AR as well as Worry and Dread (0.804); AM and Before-Test (0.654), AM and Time Management (0.608), AM and During-Test (0.623), AM and After-Test (0.589), AM and Incapacity (0.662), and AM as well as Worry and Dread (0.693).

**Table 4:** The correlation coefficients between the SA, AR, AM, T-TS, and TA subfactors

	SA	AR	AM	Before test	Time management	During test	After test	Incapacity	Worry and dread
SA	1.000								
AR	0.586^a^	1.000							
AM	0.703^a^	0.686^a^	1.000						
Before-Test	0.924^a^	0.776^a^	0.654^a^	1.000					
Time Management	0.875^a^	0.724^a^	0.608^a^	0.554^a^	1.000				
During-Test	0.851^a^	0.756^a^	0.623^a^	0.538^a^	0.638^a^	1.000			
After-Test	0.824^a^	0.706^a^	0.589^a^	0.609^a^	0.598^a^	0.612^a^	1.000		
Incapacity	0.932	0.815^a^	0.662^a^	0.732^a^	0.684^a^	0.729^a^	0.641^a^	1.000	
Worry and Dread	0.955^a^	0.804^a^	0.693^a^	0.748^a^	0.789^a^	0.832^a^	0.611^a^	0.556^a^	1.000

^a^Correlation is significant at the 0.01 level (2-tailed)

## Discussion

This research intended to investigate the impact of the SA, AR, and AM on T-TS and TA management in academic settings. To accomplish this goal, an empirical study was carried out with EFL students enrolled in English language institutions learning English via Telegram. The analysis of the data revealed that enhancement in SA, AR, and AM enables learners to pass their tests skillfully and manage their possible test anxiety. These findings underlined the crucial part that social media, specifically the Telegram app, plays in improving students’ mental and emotional health, and the suggested model (Fig. 1) was confirmed. In the following lines, the study finding is elaborated in detail as follows:

Taking into consideration the first and the second research questions, which were “RQ1:

Can EFL learners’ SA provide insight into their T-TS in online classes? and RQ2: Can EFL learners’ SA provide insight on their TA in online classes?”, the findings of this exploration show that SA can play a moderator function in T-TS and TA management (model 1 & model 2). It is possible to infer that how students approach self-evaluation may help them build or restore a positive self-image and a strong sense of self-worth, which in turn helps to reinforce the accomplishment of language learning objectives. Furthermore, SA helps learners cultivate an internalized confidence in their abilities and a perception of effectiveness in their skills to complete assignments effectively (Ismail & Heydarnejad, 2023). As Aydin et al. (2006) highlighted, test anxiety has a significant impact on the attainment and assessment of students. As a result, training programs for teachers and examiners can be useful so that they can better handle test objectives.

Additionally, model 2 demonstrated that SA successfully predicted the skillful management of test and self-improvement goals of EFL learners. It was also demonstrated that SA successfully predicted the self-improvement of learners in language assessment. Model 2 emphasized the significance of having learners participate in SA, which in turn encourages students to describe the causes and goals behind their accomplishment results in terms of raising their level of skill and the amount of work they put forth. The numerous theoretical viewpoints and empirical investigations on SA have highlighted the significant implications that SA has in the process of language assessment (e.g., Heydarnejad et al., 2022; Jahara et al., 2022).

Following the results of this research, AR was useful in fostering T-TS and warding off TA among EFL learners. Based on the outcomes of prior research (e.g., Khadem et al., 2017; Shafee Rad & Jafarpour, 2022), AR boosted student enthusiasm and performance in the classroom. The result is consistent with the motivation/demotivation theory debate and the notion of resilience. It may be deduced that AR helps college students become more aware of and creative about methods for controlling their emotions and coping with test pressures. According to the self-determination theory (Martin, 2006), strengthening one’s self-awareness increases educational objectives, educational satisfaction, resilience, and cooperation in-class activities. It means that in the face of adversity, EFL students with a high AR can respond constructively by setting attainable objectives and working hard to assimilate and adjust to the group standards and interpersonal situations of their surroundings.

It is worth highlighting that TA depends on the factors that learners experience in their classroom. When they feel at ease during learning processes and motivated to do their learning tasks, they would not experience TA, or the depth of anxiety would decrease a lot (model 1 & model 2). Based on the opportunities provided by the Telegram app, learners can easily communicate, and the anxiety of being judged by their peers would decrease. This outcome is in accordance with Esmailzade Ashini et al. (2020), who concluded that the Telegram application could help learners manage their anxiety in language classes and assessments. Students are less concerned about being evaluated by their peers as a consequence of the alternatives offered by the Telegram app, which will make it simple for them to communicate with one another. The Telegram app can lessen the pressure that comes with being evaluated. This result is in line with the results of Zhao et al. (2022), who concluded that the Telegram program may help students better regulate their anxiety while attending language classes.

The results of the fifth and sixth research questions revealed that AM played a significant role in increasing EFL learners’ T-TK and decreasing TA. That is, AM inspires students to act with vigor and build a good self-image and gives them assistance and engagement (model 1 & model 2). Both Dörnyei et al. (2016) and Al-Hoorie et al. (2022) state that successful second language acquisition is assured when the learners have the commitment and motivation necessary to accomplish their objectives. Self-motivated language students are in search of new methods to improve their skills, and barriers do not deter them. More significantly, test anxiety and language competence are opposites (Khoshhal, 2021); this means that language efficiency will rise if TA is reduced. When the process of learning a language is hampered by TA, AM can aid language learners in easing the tension and worry that they are experiencing.

## Conclusion and implications

In a nutshell, the purpose of this inquiry was to uncover the connection between SA, AR, AM, T-TS, and TA management in the EFL context. In this respect, a model was hypothesized and then verified using CFA and SEM in the proper manner. Furthermore, this study was to provide the spotlight on the advantages that Telegram-based training may bestow to language assessment when applied in an EFL environment. The obtained data provide support for the proposed model and reflect the predictive capacity of SA, AR, and AM, respectively.

In light of the results of the research, there are a number of pedagogical recommendations that may be made. Implementation of MALL into the curriculum has the potential to boost and extend teachers’ and student access to course data and foster LOA whenever and wherever, even outside of the classroom, as the outcome of the swift development of emerging technologies and the need for hosting online and virtual courses. This enhancement is of great importance in the realm of language learning and assessment. As it was displayed in this the current research, language learners felt more secure and less anxious. As such, educators need to learn the digital literacy necessary to use MALL platforms, notably the Telegram app, for the purpose of language instruction and assessment. It is necessary for both instructors and language learners to acquire an awareness of the self-aid conceptions and the attributions such constructs have on their well-being and LOA enhancement. It is important for the academic courses to either directly or indirectly include training in the relevant methods.

It is recommended that those responsible for developing academic curricula and materials make changes to such materials and take these results into consideration when working on other academic fields. It is advised that policymakers, curriculum designers, material producers, test developers, and language teachers give some thought to language instruction based on MALL and teaching self-aid practices. This will ensure the academic success of the learners, LOA, and, more significantly, the well-being of society. In addition, practical tactics that may be used to cultivate and put into practice SA, AR, AM, and T-TS should be introduced into higher education. Language teachers may get the necessary training via pre-service and in-service training courses, respectively.

Although this research provides interesting insights into the topic in question, it faces some limitations, which need to be addressed. To get things started, all of the learners who participated in our investigation were aged between 17 and 23 with similar English proficiency. In forthcoming studies, it may be possible to evaluate the correlations between SA, AR, AM, T-TS, and TA management in a variety of educational environments and among students majoring in a variety of different fields of study. In addition, due to the fact that all of the information gleaned from this study was obtained via the self-reporting of the learners on questionnaires, the generalizability of the findings is under question. In future research, incorporating qualitative and quantitative methods may provide more thorough findings. This study was limited to telegram-assisted language learning. As a future research avenue, it is suggested to investigate the effects of applying other apps in language learning enhancement and psychological health of the learners. Moreover, an additional survey is suggested to triangulate the study findings and to undertake a comprehensive analysis of the degree to which demographic factors of the students may have an impact on the interrelationship among SA, AR, AM, T-TS, and TA management.

## Data Availability

The authors state that the data supporting the findings of this study are available within the article.

## Abbreviations

EFL: English as a foreign language
LOA: Learning-oriented assessment
SA: Self-assessment
AR: Academic resilience
AM: Academic motivation
T-TS: Test-taking skills
MALL: Mobile-assisted language learning
SDT: Self-determination theory
ACT: Attentional control theory
CSAQ: The core of the self-assessment questionnaire
ARS: The Academic Resilience Scale
T-TSS: The Test-Taking Skills Scale
WTAS: The Westside Test Anxiety Scale
AMS: The Achievement Motivation Scale
SEM: Structural equation modeling
CFA: Confirmatory factor analysis
LISREL: Linear structural relations
RMSEA: The root-mean-squared error of approximation
CFI: The comparative fit index
NFI: The normed fit index
